# Supplementary figures and images for: Dissecting peripheral protein-membrane interfaces
Source: PLoS Comput Biol. 2022 Dec 14;18(12):e1010346. doi: 10.1371/journal.pcbi.1010346 (PMC9797079; doi:10.1371/journal.pcbi.1010346)

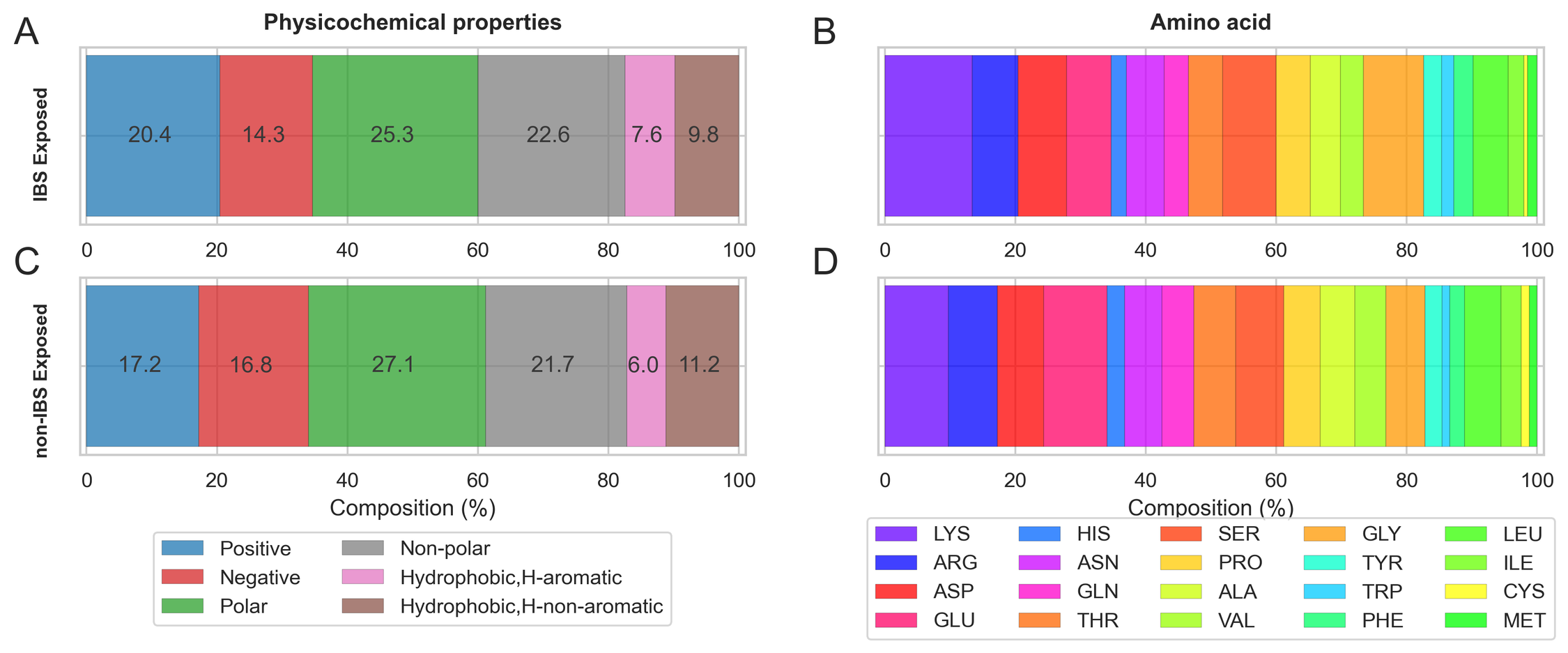

Supplement: S1 Fig — Composition is calculated across all superfamilies and grouped by (A,C) amino acid properties (positive, negative, polar, nonpolar) and (B,D) the 20 amino acids types for amino acids belonging to the exposed IBS surface (A,B) and exposed non IBS surface (C,D). (TIF) [file pcbi.1010346.s001.tif]

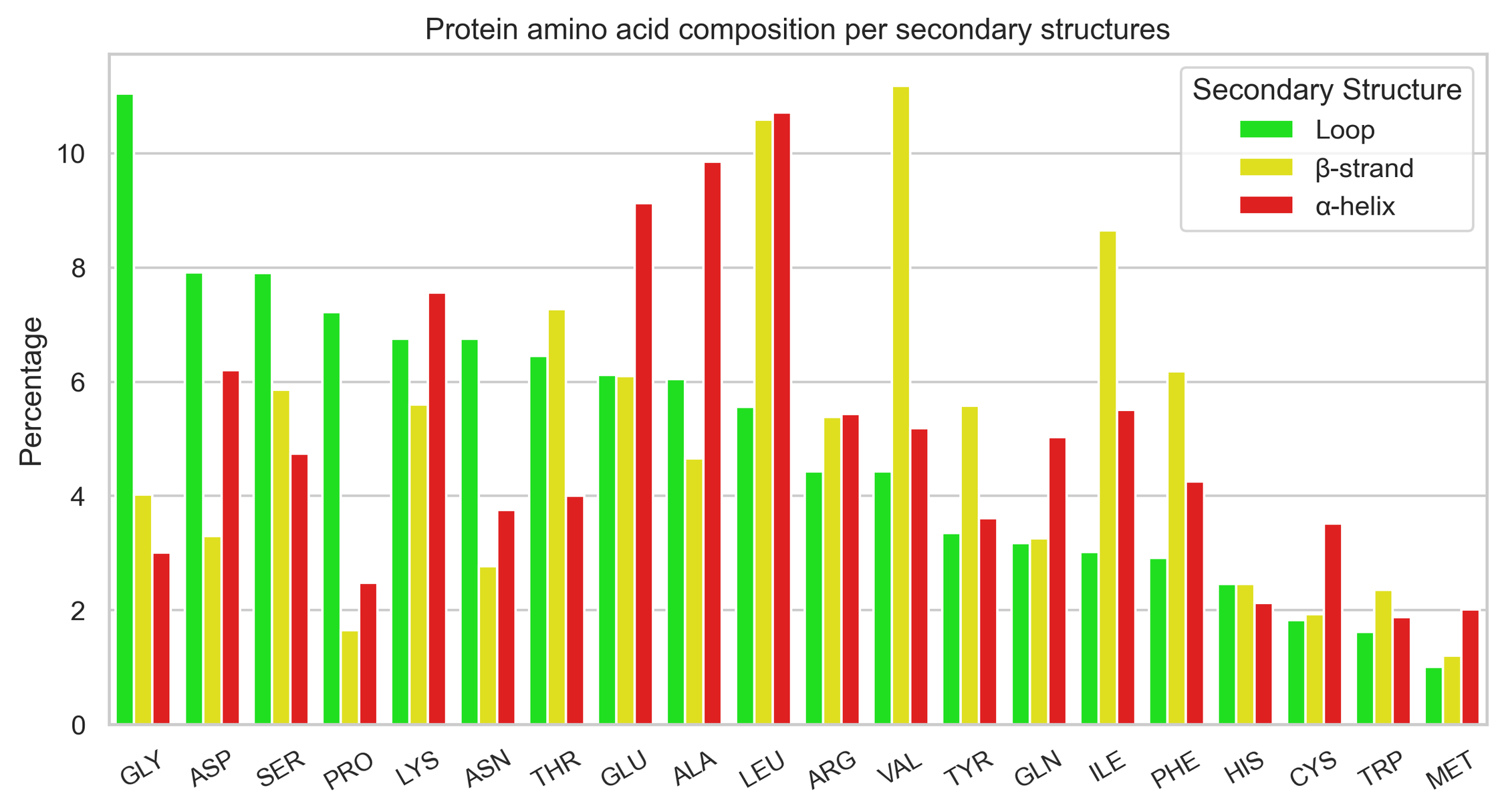

Supplement: S2 Fig — (TIF) [file pcbi.1010346.s002.tif]

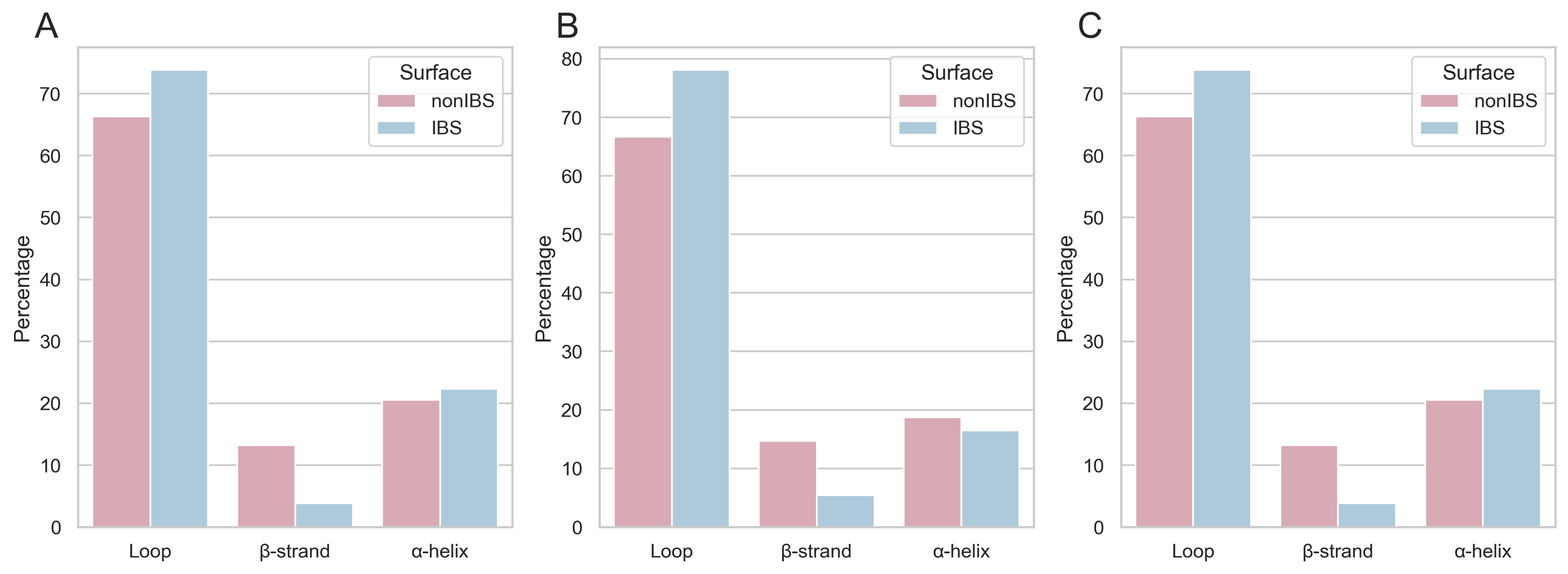

Supplement: S3 Fig — Secondary structures composition of (A) the environment of hydrophobic protrusions in protein with hydrophobic protrusion at their IBS, (B) all protrusions and (C) their environment in proteins without hydrophobic protrusions at their IBS. (TIF) [file pcbi.1010346.s003.tif]

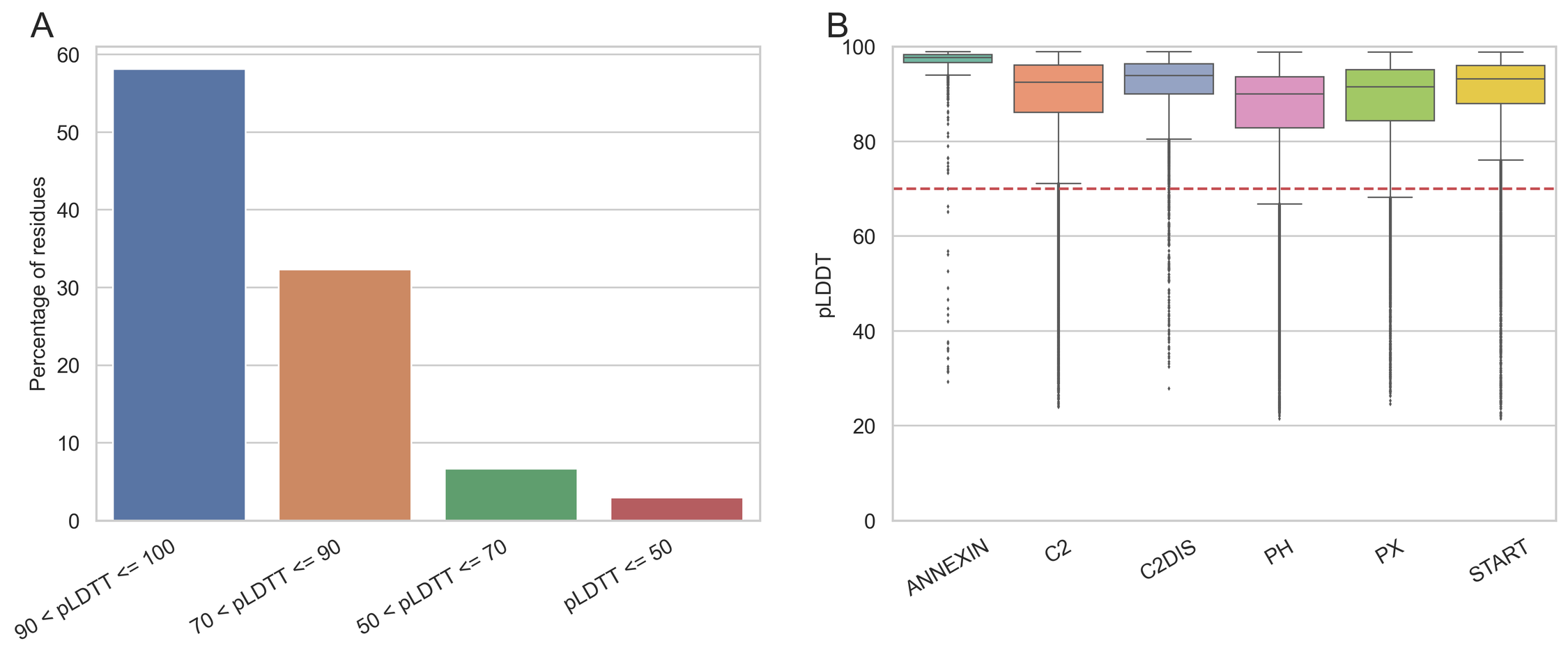

Supplement: S4 Fig — (A) Distribution of the pLDDT score per quality range. (B) Alphafold score per residue. The horizontal red dashed line represents the threshold at 70 for which the region may be unstructured in isolation according to Alphafold authors [31]. (TIF) [file pcbi.1010346.s004.tif]

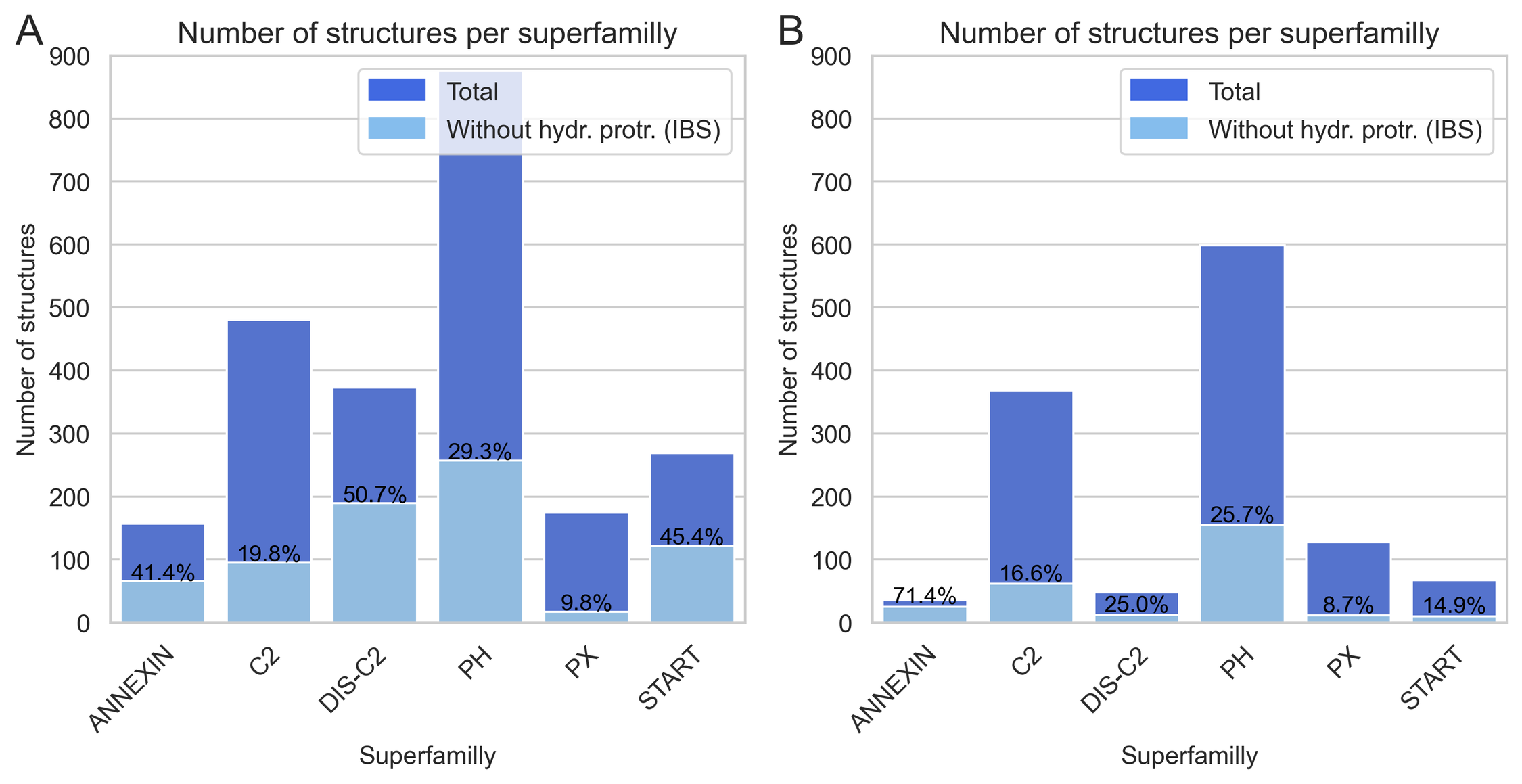

Supplement: S5 Fig — Total number of structures in each of the superfamilies in the extended dataset (A) CATH and Alphafold database and (B) Alphafold models only, with their respective percentage of structures without hydrophobic protrusions in the IBS (light gray). (TIF) [file pcbi.1010346.s005.tif]

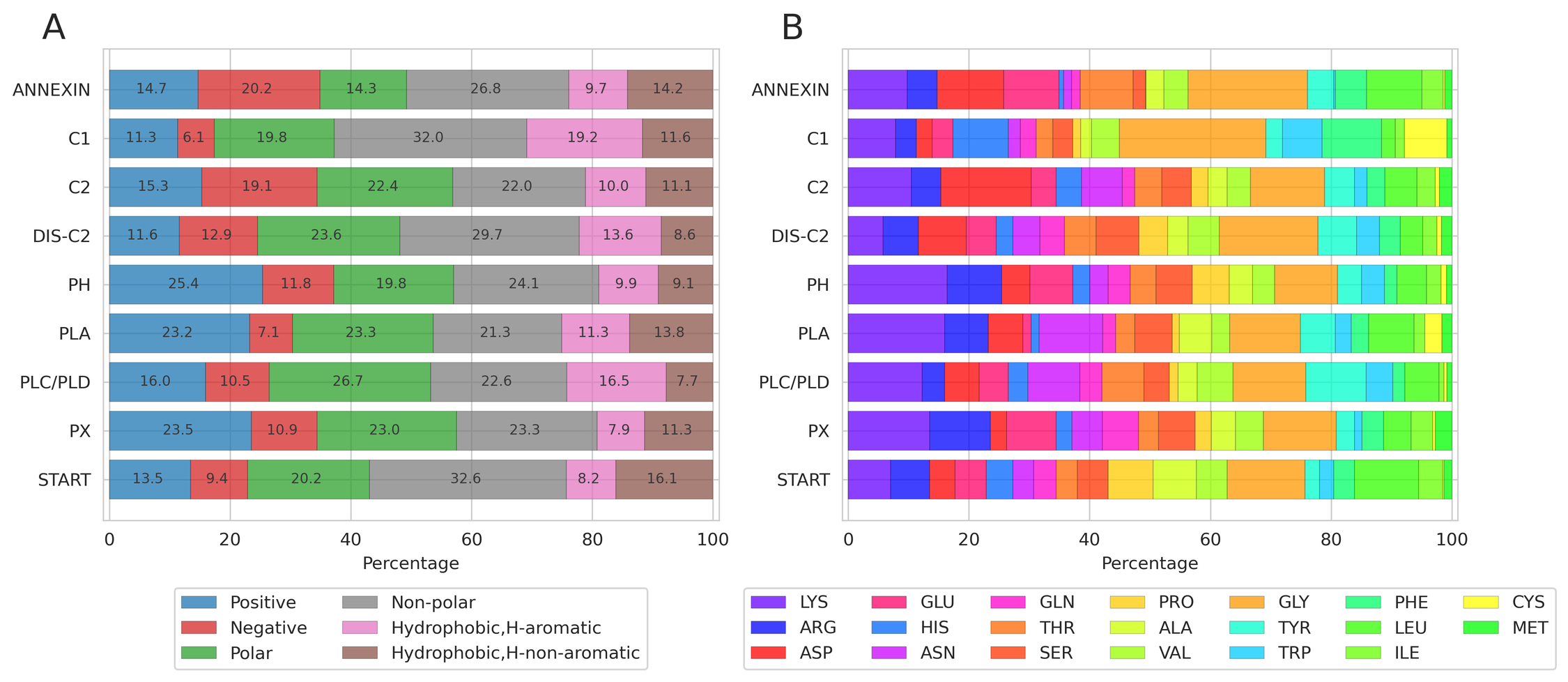

Supplement: S6 Fig — Values are stacked per superfamily and per amino acid type (A) and name (B) colored according to the “shapely” rastop color scheme. (TIF) [file pcbi.1010346.s006.tif]
