# Supplementary material for: Dissecting peripheral protein-membrane interfaces
Source: PLoS Comput Biol. 2022 Dec 14;18(12):e1010346. doi: 10.1371/journal.pcbi.1010346 (PMC9797079; doi:10.1371/journal.pcbi.1010346)
Supplement: Attachment — Submitted filename: answer_reviewers.docx [file pcbi.1010346.s012.docx]

Answer to reviewers

(Tubiana et al., Dissecting peripheral protein-membrane interfaces)

**Reviewer #1**

In this study, Tubiana et al build on their previous published work [Fuglebakk et al 2018], in the same journal. They characterise the basis of interfacial binding sites (IBS) for a range of peripheral membrane proteins (PMPs), using an impressive number of proteins for their analysis. In particular, the study is able to take advantage of the recent release of a large number of AlphaFold (AF) structures, so is very timely.

We truly appreciate the reviewer’s positive opinion of our work and thank him for insightful questions and suggestions, as well as for revealing his identity.

In general, the study is well conducted and rigorous, with some interesting insights. I recommend publishing with a few small changes:

Larger comments:

1. Firstly, I’m a little unclear on exactly how the IBS are determined. It’s stated that the structural database includes known IBS, but seeing as this is an integral part of the analysis here, it would be good if this were expanded. If, for instance, the structural IBS were predicted using bioinformatics, is there a risk that the analyses here are biased by previous assumptions? Similarly, how were the IBS for the AF models determined?

We acknowledge that this should be more clearly explained in the manuscript. The IBS was determined in the same way for the experimentally-resolved structures and for the AF models. The protocol is described in the methods section in the paragraph “2. Interfacial Binding Site selection, IBS and nonIBS datasets”. We expanded that paragraph:

*“Identification of the IBS in each family was based on the literature [3,27,55,76–78], and on visual inspection of the representative structures (listed in Table 2) and their superimposition with other domains in the same superfamily. The same protocol was used for the experimentally-determined structures and the AlphaFold models.*

*We used the literature to identify the secondary structure elements (SSE) (eg loops or short amphipatic helices) involved in membrane binding for each representative domain (Cf Figure 1 and Table 2). This approach was chosen because experimental data on IBS individual amino acids is sparse and likely to be too restrictive [1]; data is available for only a small subset of PMPs and when available, it is only reported for a few amino acids while in reality the region in contact with the membrane is large. SSEs are also easy to identify in a fold and therefore provide a definition of the IBS particularly suitable for analysis within superfamilies of proteins. In the absence of sufficient sequence conservation in loop regions, we rely on structure similarity to transfer the IBS annotation between superfamily members.*

*In practice we define a plane to delimit in space the SSEs identified as forming the IBS, and the rest of the protein; the plane is defined by three amino acids positioned on the relevant SSEs (cf. reference amino acids in Table 2). We defined this plane for one representative protein in each superfamily (cf. representative structure ID in Table 2). All structures within a superfamily were then pairwise aligned with cath.superpose and the sequential structure alignment program for protein structure comparison (SSAP) [79]. The plane defined on the representative domain was used to delimit the IBS and non-IBS regions for each domain in the same superfamily (Fig 1). Gathering this annotation for all domains in the datasets lead to two sets of amino acids coined the IBS and nonIBS datasets. We provide these datasets as Supporting Information (S2 File).* ***“***

We have also added an explanation in the Introduction section and moved what was Figure 9 to the Introduction where it now is Figure 1 and shows the representatives for the 9 domains and their IBS.

Modified text in Introduction:

*“Here we circumvent that limitation by selecting a dataset of protein domains for which the membrane-binding region is reported for several domains in the superfamily and that there exists sufficient structural data to derive statistics from the dataset. We effectively assume that the membrane-binding region within a superfamily consists of the same elements of the fold for all domains in that superfamily, a common assumption in functional annotation of protein domains.”*

In the discussion section (last paragraph), we explain why our results support the protocol used to determine the IBS regions:

*“Our observations within superfamilies are in good agreement with the known function of the domains, indicating that our strategy captures the IBS correctly. It is important because the strategy we used to build the datasets is relying on a number of approximations necessary to gather a large set of PMP structures.*

*…*

*…*

*Altogether the analyses of our datasets reveal characteristics of IBS that are in excellent agreement with what we know of PMP membrane binding mechanisms and functions. As such our results support the approach used to collect the IBS PMP datasets which are a useful resource that can be developed further, given the lack of experimental structural data on PMP-membrane interfaces.****“***

2. The analysis is done on completely rigid structures, solved in most cases I presume in a soluble state. I feel that this represents a possible limitation: if the IBS changes upon membrane binding (especially seeing as many sites contain loops) might this skew or change the data at all? For instance, could hydrophobic residues become more prominent upon binding? It would be good if this was addressed in the discussion.

This is indeed a very good point and a limitation of the study which deserves to be addressed in the discussion. We have added the following in the last paragraph of the discussion:

“*Another approximation is that the study is performed on X-ray structures of the protein in their soluble state as this is the only state we have structural data for. Some PMPs might undergo structural changes upon membrane binding and, as a consequence, buried amino acids could become exposed. We verified that including the buried amino acids in the neighborhood of protrusions (Fig.5 & 7) did not modify the outcome of the analysis (data not shown). This indicates that hydrophobes are mostly already quite exposed in the soluble forms of PMPs, which makes sense to take advantage of the hydrophobic effect.*”

3. Whilst the AF models are a really nice addition to the story, is there a risk that the relatively low accuracy of the AF side chains might skew an analysis of “protrusions”? Could this explain the difference between the AF and non-AF structures in terms of hydrophobic protrusions at the IBS (20 vs 39%)?

This is a very good point but we have no data indicating that this is the case. In section 5.1 we compared 219 domains that were present both in our dataset of experimentally-determined structures and in the dataset of predicted AlphaFold models. For 6 of the 9 superfamilies, we could not find any statistical difference in terms of the number of hydrophobic protrusions at the IBS (Table 1, P-values of Wilcoxon rank test > 0.05). The 3 superfamilies for which there was a significant difference (C1, PLA, PLD) were excluded from analysis of hydrophobic protrusions (S5 Fig).

4. As an additional thought (not necessary for publication, but interesting) it is known that some domains bind to membrane with far higher affinities than others. Can this knowledge feed into the analysis here? i.e. do the proteins with no hydrophobic protrusions at the IBS fall into families which typically have lower binding affinities? Or do PMPs with more Arg have higher affinities, which is why many PMPs have fewer Arg (so they don’t bind too tightly?)

This is an exciting thought. We have not collected affinity data for proteins in our dataset so it is difficult to address this question quantitatively here, but in a publication from 2007 in BMC Structural Biology, Andrei Lomize and co-workers collected experimentally determined membrane affinity data from a small dataset of 34 proteins (Table 4 in their paper^[1]^). The proteins were sorted in three categories, those that bind predominantly using nonspecific hydrophobic association, those with predominantly nonspecific electrostatic association and those binding specifically of lipid ligands (lipid clamps). Proteins binding with predominantly non-specific electrostatic have lower experimental affinities but with there are only two proteins in that category so it is difficult to generalize. Lipid clamps and proteins binding with predominantly hydrophobic association have comparable range of affinities, higher than the two with predominantly non-specific electrostatic.

It would be interesting to evaluate this at the superfamily level too if one could find enough available affinity data that is experimentally-determined. Accurate computed ΔG_bind_ could be used as well but high-resolution data is rare and the calculated free energies of binding to a membrane (ΔG_bind_) available in the OPM database show a poor correlation with experimental data according to the authors themselves^1^.

We have now added a sentence in the Discussion:

*“It is unclear yet how the balance between hydrophobic effect and electrostatics relate to the affinity of the domains for membranes but it was suggested – albeit on a very small dataset – that proteins binding with predominantly non-specific electrostatic might have lower experimental affinities than those binding with predominantly hydrophobic mechanism.”*

5. Smaller comments:

- The number of AF models used is reported to be 1423 in the intro, but 1194 in the abstract and discussion. Is this a discrepancy?

This typo is now corrected, the correct number is 1194.

- The figures were a little blurry and the graph labels were often difficult to read. I recommend larger labels and higher resolution for the final submission.

We improved the resolution of the figures inserted in the manuscript and increased the size of the graph labels.

- The provided GitHub link doesn’t work (pepr2ds). I think I found it, but this should be changed in the manuscript.

We apologize for this, the link has been replaced with the correct one.

- P5 line 108: “we extent this dataset” > “we extend this dataset”

Corrected.

**Reviewer #2:**

The work is technically well done and provides important data and models on amino acids and regions of proteins and domains that interact with lipid membranes.

The main shortcoming of the work is the novelty and significance. The peripheral proteins and domains that bind lipid membranes have been studied in great detail both computationally and experimentally. The work presented herein is technically solid and important but doesn't make a significant advance in new information on lipid binding amino acids or composition of the interfacial binding surface amino acids. Hydrophobic and aromatic amino acids have been identified extensively in previous studies. This is good as the computational work and models confirm what has previously been measured/known but doesn't advance the field in significantly new ways.

While we appreciate the reviewer’s comment on the solidity and importance of our work, we respectfully disagree with their statement about its novelty and significance. It is correct that hydrophobic amino acids and aromatics have been earlier identified as being important for peripheral membrane binding, and we do not claim this as a main result of our work.
 We highlight below four key results that we believe constitute advances in the field:

(1) We contribute to the field a large curated PMP dataset and an analysis pipeline that can be used and further built upon for future studies of PMPs properties.
(2) IBS of peripheral membrane proteins contain significantly more lysines than arginines.
(3) *Protruding* hydrophobes are a landmark of ca. 2/3 of PMPs.
(4) IBS of peripheral membrane proteins contain significantly more glycines than the rest of their surface.

**Reviewer #3**:

This article presents the collection of a dataset of peripheral membrane proteins (PMPs) belonging to 9 superfamilies. For each PMP, the authors characterized the interfacial binding site (IBS) and split the dataset into two datasets: the amino acids in the IBS and the rest amino acids. The authors analyzed the amino acid distribution in these datasets and applied a mathematical model of hydrophobic protrusions that the authors present in “Fuglebakk et al. PLoS Comput Biol 2018”. New patterns were discovered in the IBS, such as the over-representation of glycine amino acids and the preference for lysine over arginine amino acids. I believe that this new PMP dataset will contribute to the creation of novel machine learning algorithms and PMP prediction tools. I would therefore recommend the publication of the manuscript.

We thank the reviewer for their positive comments on our work and thank them for insightful questions and suggestions.

However, some major comments must be addressed to improve the quality of the manuscript:

1) The manuscript is about “Dissecting peripheral protein-membrane interfaces” but as it is also mentioned by the authors “the IBS for each domain is broadly defined and is likely to include more amino acids than the ones actually interacting with the lipids”. I understand that it is not possible to manually dissect ~2500 proteins but since other researchers might use this dataset for training machine learning algorithms or validating prediction models, more emphasis should be given to removing from the IBS the amino acids that do not contribute to the protein-membrane affinity.

We are grateful that this is being brought to our attention as this gives us the opportunity to better explain the goal and scope of our study. By design, our study focuses on the whole region in contact with the membrane, and not solely on the amino acids that are known or predicted to contribute to the protein-membrane affinity, which is a valid strategy but already used by others (e.g. Chatzigoulas and Cournia, Briefings in Bioinformatics, 2022; Lomize et al., BMC Bioinformatics, 2007).

First of all, experimental data is available for only a few (50-60) proteins and the low sequence conservation in loops prevents to transfer that information between all domains in a superfamily. Moreover it is nearly impossible to identify amino acids that do not contribute to the protein-membrane affinity. Hotspots of the protein-membrane interactions are identified by measuring the affinity of the wild type protein and of a few mutants chosen at the supposed IBS. Such studies do not systematically test the dozen or more of amino acids located in the membrane-binding region. They thus cannot rule out that amino acids others than the ones actually probed are contributing. Defining the IBS in this way is too limiting for the type of analysis we conducted, ie describing the properties of the whole region of the protein in contact with the membrane. That approach though is very useful for other purposes (ie development of methods to predict hotspots). The approach we have chosen is different, as we select all amino acids located in the region known to be involved in membrane binding. For example, this allows us to identify glycines in protein-membrane interfaces, yet we know that they are not interacting with the lipids as they do not have a sidechain.

We modified the Discussion and added the following:

*“One approximation is that the IBS for each domain is defined as a region on the protein surface instead of a collection of experimentally identified hotspots [68] because (1) such data is rare (available for about 50 proteins [68]) and (2) studies identifying hotspots are not systematic alanine-scanning so they are unlikely to map all amino acids involved in membrane-binding. Our strategy is justified by the fact that the protein-membrane interface is not restricted to a few hot-spots amino acids as in protein-ligand interfaces. Instead, the membrane is a surface which might get in contact with a fairly large patch of amino acids of a PMP, also beyond those that inserts in the membrane, and our study aims at characterizing the whole region.”*

We have also modified the methods section to give a clearer explanation of how the IBS is defined:

“*Identification of the IBS in each family was based on the literature [3,27,55,76–78], and on visual inspection of the representative structures (listed in Table 2) and their superimposition with other domains in the same superfamily. The same protocol was used for the experimentally-determined structures and the AlphaFold models.*

*We used the literature to identify the secondary structure elements (SSE) (eg loops or short amphipatic helices) involved in membrane binding for each representative domain (Cf Figure 1 and Table 2). This approach was chosen because experimental data on IBS individual amino acids is sparse and likely to be too restrictive [1]; data is available for only a small subset of PMPs and when available, it is only reported for a few amino acids while in reality the region in contact with the membrane is large. SSEs are also easy to identify in a fold and therefore provide a definition of the IBS particularly suitable for analysis within superfamilies of proteins. Moreover, in the absence of sufficient sequence conservation in loop regions, we rely on structure similarity to transfer the IBS annotation between superfamily members.*“

2) Most of the analysis is performed on the mathematical model of hydrophobic protrusions that the authors present in “Fuglebakk et al. PLoS Comput Biol 2018” instead of performing analysis on the dataset itself. It is like the purpose of the manuscript has changed to test the model of hydrophobic protrusions in a new dataset. The only analysis performed in the dataset is the amino acid distribution in the IBS vs the nonIBS sets. More analysis between all or the solvent-exposed amino acids in the IBS vs the nonIBS would provide better insights into important properties of the IBS. For example, the secondary structure elements between the IBS and nonIBS, the amino acid conservation between the IBS and nonIBS in each superfamily, etc.

We wonder if this comment could be due to a misunderstanding. Let us explain the difference between the two studies. The hydrophobic protrusion model was proposed, tested and validated in Fuglebakk et al. (2018): we showed that hydrophobic protrusions are a pattern characteristic of the surface of about 2/3 of PMPs, by comparing PMPs to protein domains that do not bind to membranes. That conclusion was for whole protein surfaces, not restricted to IBSs. In addition we verified on a small dataset that particular hydrophobic protrusions coincided with the experimentally-determined IBS.

We state the following in the introduction:

*“In Fuglebakk et al. [1] we analysed surfaces of whole structures instead of focusing on the IBS, owing to the limited availability of experimental determination and annotation of interfacial membrane-binding sites. Here we circumvent that limitation by selecting a dataset of protein domains for which the membrane-binding region is reported for several domains in the superfamily and that there exists sufficient structural data to derive statistics from the dataset.”*

In the submitted manuscript we built a new and large dataset that allows us to compare the IBS to the rest of the surface (non-IBS). We report differences in terms of the secondary structure elements (Fig.4A, Fig.S3), the number and nature of protrusions (i.e hydrophobe and non-hydrophobe Fig.4A and Fig7A), the amino acid composition around the protrusions (Fig.5 and Fig.7B).

We thus feel that our manuscript present new results compared to our 2018 paper, and most of what the reviewer is asking is already present in our manuscript. When it comes to conservation in the IBS vs non-IBS, this is an excellent idea that we investigated. Unfortunately most IBS for the domains in our dataset are in variable loop regions and we could not get reliable sequence alignments for these regions. As a consequence it is impossible to obtain measures of conservation.

I have also some minor comments and suggestions to improve the clarity of the presentation:

1) Main text, Page 11, Lines 237-243: I cannot understand why the fact that “proteins without hydrophobic protrusions at their IBS tend to have less protrusions in their IBS in general” is significant. The authors should elaborate more on the scope of this analysis.

Apologies for the lack of clarity of this sentence. This was actually just an explanation of why we see no hydrophobic protrusions at the IBS of these proteins. We have reformulated this sentence to hopefully make it clearer:

“*The proteins without hydrophobic protrusions at their IBS have less protrusions in their IBS in general, so the absence of hydrophobic protrusions is probably just the consequence of the structure of these proteins at the IBS (ie few protrusions)*.”

2) The fact that glycine amino acids are over-represented in the IBS is interesting. Indeed, one possible explanation is that glycine amino acids provide flexibility, which helps hydrophobic loops to insert the membrane. Do you think that the absence of a side chain in glycine amino acids drives nearby hydrophobic and aromatic amino acids to be more solvent-exposed and thus it is easier for them to anchor on the membrane? If yes, you could also discuss it in the manuscript.

This is a excellent suggestion, and we agree with the reviewer. We added the following text in the Discussion about the role of glycines:

*“It might also be that their small size decreases steric hindrance and favors the presence of large hydrophobes on the protein surface.”*

3) In Table 1 the column “Number of structures in common” should be described in the caption.
Done.

4) I suggest adding colors to Figures 5B, 7A, and S4.

We added colors to those figures. Note the new numbers 6B, S4A and S5 following the changes suggested below (comment number 5).

5) In my opinion, the main text contains a lot of figures and tables. I suggest moving Figure 7 and Table 3 to the SI.

We have moved the suggested Figure and Table to the SI.

6) Main text, Page 25, Line 567. I believe there is a typo here and instead of Fig. 1, it should be Fig. 9.

Done

7) Reference 9 and reference 11 are the same.

The duplicated reference has been removed.

1. DOI: [10.1186/1472-6807-7-44](https://doi.org/10.1186/1472-6807-7-44)
